# Possible hepatotoxicity of chronic marijuana usage

**DOI:** 10.1590/S1516-31802004000300007

**Published:** 2004-05-06

**Authors:** Paulo Borini, Romeu Cardoso Guimarães, Sabrina Bicalho Borini

**Keywords:** Marijuana, Cannabinoids, Illicit drugs, Liver, Addictive behavior, Maconha, Canabinóides, Drogas ilícitas, Hepatopatias, Fígado

## Abstract

**CONTEXT:**

Hepatotoxicity is a potential complication from the usage of various illicit drugs, possibly consequent to their liver metabolism, but information on this is scarce in the medical literature.

**OBJECTIVE:**

To study the occurrence of clinical and laboratory hepatic alterations in chronic marijuana users, from the use of marijuana on its own or in association with other legal or illicit drugs.

**TYPE OF STUDY:**

transversal study

**SETTING:**

Hospital Espírita de Marília, Marília, São Paulo, Brazil

**PARTICIPANTS:**

The study was made among 123 patients interned in the Hospital Espírita de Marília from October 1996 to December 1998, divided into 3 groups: 26 (21%) using only marijuana, 83 (67.5%) using marijuana and crack, and 14 (11.4%) consuming marijuana and alcohol.

**PROCEDURES AND MAIN MEASUREMENTS:**

Patients were examined clinically with special emphasis on types of drugs used, drug intake route, age when consumption began, length and pattern of usage, presence of tattooing, jaundice, hepatomegaly and splenomegaly. Serum determinations of total proteins, albumin, globulin, total and fractions of bilirubin, aspartate (AST) and alanine (ALT) aminotransferases, alkaline phosphatase (AP), gamma-glutamyltransferase and prothrombin activity were performed.

**RESULTS:**

Among users of only marijuana, hepatomegaly was observed in 57.7% and splenomegaly in 73.1%, and slightly elevated AST (42.3%), ALT (34.6%) and AP (53.8%). The three groups did not differ significantly in the prevalence of hepatomegaly, splenomegaly and hepatosplenomegaly. The group using both marijuana and alcohol showed the highest prevalence of alterations and highest levels of aminotransferases. Mean AP levels were above normal in all groups.

**CONCLUSIONS:**

Chronic marijuana usage, on its own or in association with other drugs, was associated with hepatic morphologic and enzymatic alterations. This indicates that cannabinoids are possible hepatotoxic substances.

## RESUMO

**CONTEXTO:**

Hepatotoxidade é uma complicação potencial do uso de várias drogas ilícitas, possivelmente como conseqüência do seu metabolismo hepático. Entretanto, informações sobre tal possibilidade são escassas na bibliografia médica.

**OBJETIVO:**

Estudar a ocorrência de alterações clínicas e laboratoriais hepáticas que podem ocorrer em usuários crônicos de maconha, isoladamente ou associadas ao uso de outras drogas lícitas e ilícitas.

**TIPO DE ESTUDO:**

estudo transversal

**LOCAL:**

Hospital Espírita de Marília, Marília -SP, Brasil.

**PARTICIPANTES:**

Foram estudados 123 pacientes, internados no Hospital Espírita de Marília de outubro de 1996 a dezembro de 1998, divididos em três grupos: 26 (21%) usuários exclusivamente de maconha, 83 (67,5%) usuários de maconha e crack e 14 (11,4%) usuários de maconha e álcool.

**PROCEDIMENTOS E VARIÁVEIS ESTUDADAS:**

Os pacientes foram examinados clinicamente, com especial ênfase nos aspectos relativos aos tipos de drogas usadas e rotas de usos, idade de início do uso, tempo e padrão de uso, presença ou ausência de tatuagem, icterícia, hepatomegalia e esplenomegalia. Foram determinados os níveis séricos de proteínas totais, albumina, globulina, bilirrubina total e frações, aspartato-aminotransferase (AST), alanina-aminotransferase (ALT), fosfatase alcalina (FA), gama-glutamiltransferase e atividade da protrombina.

**RESULTADOS:**

Entre os usuários exclusivos de maconha foram observados hepatomegalia em 57,7% e esplenomegalia em 73,1% dos casos, e estavam discretamente elevadas a AST (42,3%), ALT (34,6%) e FA (53,8%). Os três grupos não diferiram significativamente nas prevalências de hepatomegalia, esplenomegalia e hepatoesplenomegalia. No grupo maconha/álcool foram observadas as maiores prevalências de alterações e níveis mais elevados das aminotransferases. Os níveis médios da fosfatase alcalina estavam acima do valor normal em todos os grupos.

**CONCLUSÕES:**

O uso crônico de maconha, exclusivo ou associado a outras drogas, associou-se a alterações morfológicas e enzimáticas hepáticas, sugerindo serem os canabinóides substâncias possivelmente hepatotóxicas.

## INTRODUCTION

Hepatotoxicity is a potential complication from the usage of various illicit drugs, possibly consequent to their liver metabolism, but information on this is scarce in the medical literature. Studies on liver damage due to chronic usage of marijuana are rare.

Marijuana usage in the form of cigarettes made from dried leaves, flowers and stalks of female *Cannabis sativa* plants has grown strongly among young Brazilians over the last decade.^1^ In the United States, after some decline in the prevalence of cannabis usage, a recent upsurge has been detected. It has been conceded that this has taken place consequent to the relaxation of social disapproval of marijuana usage, a reduction in the perception of the risks related to usage and an increase in the dissemination of pro-drug media messages.^2^ In general, marijuana is the first drug to be used. Subsequently, other drugs with stronger effects may substitute for it, or other legal or illicit drugs may start to be taken in association with it.^3^ Associations with cocaine or crack have the aim of reducing or avoiding the dysphoric effects of these two drugs, while with alcohol the aim is to increase the hallucinogenic effects of marijuana.^3^

*Cannabis sativa* contains more than 400 chemical compounds, of which about 60 are cannabinoids. The first to be isolated, and the one mainly responsible for the psychoactive properties of the plant, was delta-9-tetrahydrocannabinol.^4^ The delta-9-tetrahydrocannabinol content of the drug depend on the way it is prepared, and it is possible that other components of marijuana, whether cannabinoids or not, may enhance the effects of delta-9-tetrahydrocannabinol.^4^ After inhalation, delta-9-tetrahydrocannabinol is rapidly absorbed and distributed through the circulation. The initial metabolism takes place in the lungs and liver, with conversion to 11-hydroxy-tetrahydrocannabinol, which is more potent and crosses the hematoencephalic barrier more easily. The metabolism goes further in the liver, where 11-hydroxy-tetrahydrocannabinol is converted into many inactive metabolites. Among these is 11-nor-carboxy-delta-9-tetrahydrocannabinol, which can be detected minutes after smoking, abundantly in plasma and urine.^4^

The delta-9-tetrahydrocannabinol doses needed in order to produce effects in humans vary from 2 to 22 mg per inhalation. Tolerance is not developed to marijuana but the psychoactive effects are dose-related.^5^ Marijuana is sold by the gram, in paper packages or as ready-made cigarettes of 0.5 – 1.0 g, and smoked as such, in Brazil as well as in the Unites States.^4^ The physiological effects are similar to those of the atropine group, and the psychological effects are similar to those of alcohol.^5^

It has been demonstrated that, in neurons, cannabinoids inhibit adenylcyclase, probably through interaction with an inhibitory G protein. The enzyme would become unable to convert adenosine triphosphate into the ‘second messenger’ cyclic adenosine monophosphate.^6^ Other mechanisms besides this have been proposed: inhibition of calcium channels^7^ and activation of potassium channels^8^ or the phospholipid-inositol mechanism.^9^ Of the 60 natural cannabinoids, delta-9-tetrahydro-cannabinol is the only one that binds to a membrane receptor. This receptor is present in every cell. In binding to it, delta-9-tetrahydrocannabinol displaces its natural ligand, anandamide, and persistently disrupts the physiological signaling of the receptor. The anomalies observed clinically when delta-9-tetrahydrocannabinol is present in the cell membrane are associated with the molecular disruption of membrane signaling. Because of the disruption of this molecular mechanism by delta-9-tetrahydrocannabinol, it has not been possible to separate the adverse effects of delta-9-tetrahydrocannabinol and marijuana from their therapeutic properties.^10^

Adverse effects of chronic marijuana usage have been described upon the respiratory^11,12^ and cardiovascular^10^ systems. Questions remain regarding its effects upon reproductive systems^13^ and cellular and humoral immune systems.^14,15^ Cannabis smoke is mutagenic, *in vitro* and *in vivo*, thus also suggesting carcinogenicity.^16^

Recently, in many parts of the world, including Brazil, legalization of marijuana usage has been proposed for therapeutic purposes. Such campaigns could lead to misinterpretations, especially by non-medical communities, thereby leading to evaluations that the drug might be ‘healthful’, instead of harmful to health.

The present study is part of a larger one aimed at evaluating the hepatotoxicity of illicit drugs. Data presented here relate to the occur-rence of clinical and laboratory alterations to the liver among chronic marijuana users, when marijuana is consumed on its own or in association with other legal or illicit drugs.

## METHODS

From a total of 373 patients who were users of illicit drugs through non-parenteral routes, 323 (86.6%) were selected for study according to the principles of the Helsinki Declaration, with approval from the Ethics Committee of the Hospital Espírita de Marília and with patients’ informed consent. The other 50 patients refused to participate in the study or were excluded. The patients studied were interned in a private hospital (Hospital Espírita de Marília) from October 1996 to December 1998, the majority belonging to low socioeconomic classes.

Patients were examined clinically with especial emphasis on types of drugs used, drug intake route, age when consumption began, length and pattern of usage, blood transfusion, sexual behavior, use of medicine, acupuncture, presence of tattooing, jaundice, hepatomegaly and splenomegaly. Homosexuality, use of intravenous drugs, acupuncture and blood trans-fusion were criteria for exclusion.

Evaluation of the amount (grams) of the drug consumed daily was done through the patients’ reports and by comparing this with their information on the daily cost of acquiring the drugs (at the time of the study, one gram of marijuana was worth US$ 1.00 and one gram of crack US$ 10.00). Marijuana was consumed via aspiration of cigarette smoke and crack via ‘pipe’ smoke. Evaluation of daily alcohol consumption, in the majority of cases in the form of sugarcane distillate, was done through self-reports on amounts and frequency of usage.

Hepatomegaly was diagnosed when the liver was palpable in excess of 1.5 cm below the costal margin, to the right of the midclavicular line, with the upper level of liver mass not below the 5^th^ rib space.^17^ Splenomegaly was diagnosed not only when the organ was palpable below the coastal margin but also when the upper level of its mass, detected in Traube's space, was dislocated downwards when the patient performed inspiratory apnea.^18^ For the examination of the liver and spleen, the patients were in a supine position, lying on their right and left sides.

Venous blood samples were taken from patients after at least 8 hours of fasting, within the first 24 hours following admission, for determination of serum levels of total proteins (TP), albumin (Alb), globulin (Glob), total bilirubin (TB) and fractions, aspartate (AST) and alanine (ALT) aminotransferases, alkaline phosphatase (AP), gamma-glutamyltransferase (GGT) and prothrombin activity (PTA). No investigation of viral antigens was made. Parasitological stool examinations were negative for *Schistosoma mansoni* eggs for all patients.

This cross-sectional study relates to 123 patients who were marijuana users, separated into 3 groups: marijuana (n = 26; 21.1%), using only marijuana; marijuana/crack (n = 83; 67.5%), using marijuana and crack; and marijuana/alcohol (n = 14; 11.3%), consuming marijuana and alcohol. The other 200 consumers of illicit drugs were not marijuana users, but consumers of crack, cocaine and solvents only.

Data are presented as mean ± standard deviation and 95% confidence interval (CI). For qualitative variables, statistical comparisons were done by means of the chi- squared test (2 x n and 2 x 2 tables) or bicaudal Fisher's exact test, according to whether the expected values were higher or lower than 5, respectively. For quantitative variables, the comparisons were done by means of Student's t test, one-way variance analysis (ANOVA) or the Kruskal-Wallis test (after Bartlett's test for homogeneity of variances). Comparisons between groups were done by means of the Bonferroni multiple comparisons test. Correlation studies were conducted using simple regression analysis and Pearson's significance test. The 5% significance level was adopted. Data were processed through the Epi-Info 6.0 package.

## RESULTS

Demographic characteristics of the patients, the pattern of drug usage and liver serum level tests are presented in Table 1. The users of marijuana/alcohol were significantly older than those using marijuana/crack (p = 0.024, confidence interval, CI, 0.47 – 7.40), but not older than users of marijuana only. There was no difference in the age when marijuana usage began among the groups, but users of marijuana/alcohol had been using the drugs for a longer period than for those using marijuana/crack (p = 0.042, CI = 1.85 – 7.76). There was no difference in daily mean marijuana usage among the groups. No correlation was observed between amount and length of time of marijuana usage (r = 0.244; p = 0.230), amounts of marijuana and crack consumed (r = 0.09; p = 0.656) or amounts of marijuana and alcohol (r = 0.10; p = 0.520).

**Table 1:** Pattern of marijuana use, on its own or in association with crack or alcohol, and serum levels of liver function tests

Variables	Marijuana	Marijuana/ crack	Marijuana/ alcohol	p
	n = 26	n = 83	n = 14	

Age (years)	19.0 ± 5.1	18.9 ± 4.6	22.8 ± 6.6	0.024
Age when started drug usage (years)	14.0 ± 4.1	15.0 ± 4.2	15.2 ± 4.7	NS
Length of time of drug usage (years)	5.1 ± 4.3	3.8 ± 3.4	7.6 ± 6.2	0.042
Daily dose (g/day)	4.3 ± 3.6	6.3 ± 5.9	5.1 ± 5.6	NS
Total protein (6.0 - 8.0 g/dl)	6.9 ± 0.7	6.5 ± 1.0	6.7 ± 1.4	NS
Albumin (3.5 - 5.5 g/dl)	4.2 ± 0.8	4.0 ± 0.9	3.8 ± 0.9	NS
Globulin (1.0 - 3.0 /dl)	2.8 ± 1.0	2.6 ± 0.9	2.9 ± 1.3	NS
PA (70 - 100%)	76.3 ± 12.5	81.2 ± 17	75.0 ± 24	NS
TB (≤ 1.2 mg/dl)	0.66 ± 0.1	0.50 ± 0.3	0.77 ± 0.3	0.001
AST (4 - 36 U/ml)	38.3 ± 26.1	29.5 ± 22.4	54.1 ± 28.6	0.002
ALT (4 - 32 U/ml)	35.5 ± 30.5	24.2 ± 20.2	54.2 ± 40.1	0.005
AP (13 - 45 UI/l)	49.9 ± 29.0	52.0 ± 30.9	48.9 ± 26.1	NS
GGT (6 - 28 UI/l)	11.3 ± 7.9	9.7 ± 8.6	39.2 ± 59.5	NS
AST/ALT ratio	1.47 ± 0.9	1.48 ± 0.8	1.33 ± 0.6	NS

*PA: prothrombin activity; TB: total bilirubin; AST: aspartate aminotransferase; ALT: alanine aminotransferase; AP: alkaline phosphatase; GGT: gamma-glutamyltransferase. Unit and normal range in parenthesis. Significance (p) in ANOVA or Kruskal-Wallis tests. Not significant: NS (> 0.05).*

The mean levels of AST and ALT were above normal limits among users of marijuana alone or marijuana/alcohol, but not in the group of users of marijuana/crack. The enzyme values among users of marijuana/alcohol were significantly higher than among users of marijuana alone (AST: p = 0.002, CI = 11.93 – 22.67; ALT: p = 0.005, CI = 15.87 – 26.13). The mean levels of AP were above normal in all groups, without significant differences among them. The mean values of other variables were within normal ranges. The TB levels showed statistically significant differences between the three groups. Only the marijuana/alcohol group showed elevated GGT. The mean levels of the AST:ALT ratio were high in all groups (marijuana, 1.47; marijuana/crack, 1.48 and marijuana/alcohol, 1.34), without significant differences between them.

No correlation was observed between the amount or length of time of marijuana consumption and the serum levels of AST (r = 0.06, p = 0.77; r = 0.109, p = 0.595, respectively), ALT (r = 0.108, p = 0.60; r = 0.109, p = 0.877, respectively), AP (r = 0.185, p = 0.365; r = 0.316, p = 0.116, respectively) and GGT (r = 0.030, p = 0.818; r = 0.013, p = 0.951, respectively).

Hepatomegaly, splenomegaly and hepatosplenomegaly were detected in 57.7%, 73.1% and 46.2%, respectively, of users of marijuana on its own, with slight to moderate elevation of AST (42.3%), ALT (34.6%) and AP (53.8%) (Table 2). In the marijuana group, spleen enlargement was detectable only through percussion, while in the marijuana/crack and marijuana/alcohol groups, the spleen was palpable in three (3.6%) and one (7.1%) cases, respectively. Considering only the marijuana group, the 15 (57.7%) cases with palpable livers were: 40% soft, not painful; 33.3% soft, painful; 26.7% firm, not painful; and none were firm and painful. The liver palpation data regarding consistency and sensitivity were variable among the groups. The prevalence of hepatomegaly, splenomegaly and hepatosplenomegaly did not differ among the groups and showed no relationship with the prevalence of enzyme alterations (Table 2).

**Table 2:** Prevalence of clinical and laboratory alterations in chronic marijuana users, when used on its own or in association with crack or alcohol

Variables	Marijuana n = 26		Marijuana/crack N = 83		Marijuana/alcohol n = 14		p
	n	%	n	%	n	%	
Hepatomegaly	15	57.7	57	68.7	10	71.4	NS
Soft, not painful	6	23.1	17	20.5	3	21.4	NS
Soft, painful	5	19.2	35	42.2	4	28.6	NS
Firm, not painful	4	15.4	4	4.8	2	14.3	NS
Firm, painful	0	-	1	1.2	1	7.1	NS
Splenomegaly	19	73.1	70	84.3	11	78.6	NS
Percussion	19	73.1	67	80.7	10	71.4	NS
Palpation	0	-	3	3.6	1	7.1	NS
Hepatosplenomegaly	12	46.2	51	61.4	7	50.0	NS
Clinical jaundice	0	-	0	-	0	-	-
Albumin ↓	6	23.1	16	19.3	5	35.7	NS
Globulin ↑	12	46.2	24	28.9	5	35.7	NS
PA ↓	4	15.4	13	15.7	3	21.4	NS
TB ↑	1	3.8	0	-	1	7.1	-
AST ↑	11	42.3	25	30.1	11	78.6	0.002
ALT ↑	9	34.6	24	28.9	10	71.4	0.009
AP ↑	14	53.8	40	48.2	7	50.0	NS
GGT ↑	1	3.8	4	4.8	4	28.6	0.005

*PA: prothrombin activity; TB: total bilirubin; AST: aspartate aminotransferase; ALT: alanine aminotransferase; AP: alkaline phosphatase; GGT: gamma-glutamyltransferase. Arrows indicate increase (up) or reduction (down) in comparison with reference values. Significance (p) in chi-squared or Fisher's exact tests. Not significant: NS (> 0.05).*

The prevalence of enzyme alterations was significantly higher in the marijuana/alcohol group, in relation to the other groups: AST (p = 0.002), ALT (p = 0.009), GGT (p = 0.005) (Table 2).

The levels of aminotransferase and AP alterations were slight in all groups. There was no significant difference in mean GGT levels among groups, in spite of the elevation shown by the marijuana/alcohol group (Table 3).

**Table 3:** Laboratory alterations in chronic marijuana users, when used on its own or in association with crack or alcohol

Variables	Marijuana n = 26	Marijuana/crack N = 83	Marijuana/alcohol n = 14	p
Albumin ↓	3.3 ± 0.2	3.0 ± 0.7	3.0 ± 0.3	NS
Globulin ↑	3.7 ± 0.3	3.7 ± 0.6	4.4 ± 0.7	NS
PA ↓	56.3 ± 5.2	53.6 ± 16.6	41.3 ± 25	NS
TB ↑	1.2 ± 0	-	1.3 ± 0	-
AST ↑	63.2 ± 21.8	58.4 ± 16.6	63.7 ± 23.3	NS
ALT ↑	67.3 ± 31.4	50.0 ± 16.3	69.1 ± 37.4	NS
AP ↑	71.1 ± 19	73.6 ± 30.6	69.3 ± 21.3	NS
GGT ↑	29.0 ± 0	38.5 ± 8.7	114.8 ± 67.2	NS

*PA: prothrombin activity; TB: total bilirubin; AST: aspartate aminotransferase; ALT: alanine aminotransferase; AP: alkaline phosphatase; GGT: gamma-glutamyltransferase. Arrows indicate increase (up) or reduction (down) in comparison with reference values. Significance (p) in ANOVA or Kruskal-Wallis tests. Not significant: NS (> 0.05).*

The effects of tattooing are depicted in Tables 4 and 5. The prevalence of ALT alterations was significantly higher in the non-tattooed subgroup of the marijuana group (p = 0.02), while in the marijuana/crack group there was high prevalence of globulin alterations in the non-tattooed subgroup (p = 0.03) and AST alterations in the tattooed subgroup (p = 0.05) (Table 4).

**Table 4:** Prevalence of clinical and laboratory alterations in patients with and without tattooing

Variables	Marijuana tattooing					Marijuana/crack tattooing					Marijuana/alcohol tattooing				
	yes n = 11		no n = 15			Yes n = 32		no n = 51			yes n = 5		no n = 9		
	n	%	n	%	p	n	%	n	%	p	n	%	n	%	p
Hepatomegaly	5	45.5	10	66.7	NS	23	71.9	34	66.7	NS	4	80.0	6	66.7	NS
Splenomegaly	10	90.9	9	60.0	NS	26	81.3	44	86.3	NS	4	80.0	6	66.7	NS
Albumin ↓	4	36.4	2	13.3	NS	5	15.6	11	21.6	NS	2	40.0	3	33.3	NS
Globulin ↑	7	63.6	5	33.3	NS	6	18.8	18	35.3	0.03	3	60.0	2	22.2	NS
PA ↓	2	18.2	2	13.3	NS	5	15.6	8	15.7	NS	1	20.0	2	22.2	NS
AST ↑	4	36.4	7	46.7	NS	14	43.8	11	21.6	0.05	5	100	6	66.7	NS
ALT ↑	1	9.1	8	53.3	0.02	12	37.5	12	23.5	NS	5	100	5	55.5	NS
AP ↑	6	54.5	8	53.3	NS	14	43.8	26	51.0	NS	2	40.0	5	55.5	NS
GGT ↑	0	-	1	6.9	NS	3	9.4	1	2.0	NS	1	20.0	3	33.3	NS

*PA: prothrombin activity; AST: aspartate aminotransferase; ALT: alanine aminotransferase; AP: alkaline phosphatase; GGT: gamma-glutamyltransferase. Arrows indicate increase (up) or reduction (down) in comparison with reference values. Significance (p) in chi-squared or Fisher's exact tests. Not significant: NS (> 0.05).*

**Table 5:** Laboratory alterations in patients with and without tattooing

Variables	Marijuana tattooing			Marijuana/crack tattooing			Marijuana/alcohol tattooing		
	yes n = 11	no n = 15	p	Yes n = 32	no n = 51	p	yes n = 5	no n = 9	p
Albumin ↓	3.3 ± 0.1	3.3 ± 0.2	NS	3.4 ± 0.1	2.8 ± 0.8	0.01	3.0 ± 0.4	3.0 ± 0.3	NS
Globulin ↑	3.4 ± 0.3	4.0 ± 0.7	NS	3.3 ± 0.2	3.9 ± 0.6	0.01	4.7 ± 0.4	4.0 ± 1	NS
PA ↓	55.5 ± 7.8	57.0 ± 4.2	NS	52.4 ± 11	54.4 ± 20	NS	63.0 ± 0	56.5 ± 23	NS
AST ↑	50.3 ± 7.7	70.5 ± 24	0.01	62.0 ± 15	53.8 ± 17	0.02	55.2 ± 9.4	70.8 ± 29	NS
ALT ↑	65.0 ± 0	67.6 ± 34	NS	48.7 ± 20	51.2 ± 12	NS	60.2 ± 41	78.0 ± 35	NS
AP ↑	68.2 ± 20	73.3 ± 19	NS	70.6 ± 25	75.2 ± 34	NS	85.5 ± 13	62.8 ± 21	NS
GGT ↑	-	29.0 ± 0	-	35.7 ± 8	47.0 ± 0	-	38.0 ± 0	140.0 ± 5	-

*PA: prothrombin activity; AST: aspartate aminotransferase; ALT: alanine aminotransferase; AP: alkaline phosphatase; GGT: gamma-glutamyltransferase. Arrows indicate increase (up) or reduction (down) in comparison with reference values. Significance (p) in Student's test. Not significant: NS (> 0.05).*

The AST levels (p = 0.016, CI = 4.64 – 35.76) were significantly raised in the non-tattooed subgroup of the marijuana group. In the marijuana/crack group, the albumin levels (p = 0.01, CI 0.32 - 0.88) and AST levels (p = 0.02, CI 0.9 - 15.5) were significantly raised in the tattooed subgroup, while globulin levels (p = 0.01, CI 0.38 - 0.82) were raised in the non-tattooed subgroup (Table 5).

## DISCUSSION

Various authors have reported difficulties in forming groups of patients using only marijuana. Users of marijuana frequently use alcohol and other illicit drugs, crack or cocaine in association with the marijuana, with the aim of increasing or reducing the euphoric or dysphoric effects of one of the drugs. Some authors have reported that patients mentioned that alcohol reduced their usual “high” state reached with the consumption of marijuana on its own,^19^ while others have observed the opposite: that alcohol increased the hallucinogenic effects.^3^ In relation to the association with cocaine or crack, the use of marijuana immediately after cocaine or crack has the aim of reducing the dysphoria (“*nóia*” or paranoia, in the users’ jargon) that they cause.^3^ We observed the same difficulties in reaching an adequate group size for users of marijuana only, due to the high prevalence of concomitant utilization of alcohol and crack, which required the examination of a large ensemble of illicit drug users.

Knowledge of liver physiopathology related to illicit drugs is still scarce. Until 1969, there was no reference in the medical literature to human marijuana hepatotoxicity. The extensive report of the Indian Hemp Commission in the nineteenth century^20^ had not revealed any occurrence of liver affection among hashish users: in hashish, the delta-9-tetrahydrocannabinol content is higher than in marijuana. Also in 1969, Kew et al.^19^ re-ported on 12 chronic marijuana users, of whom three (25%) showed evidence of liver degenerative processes in biopsy fragments. In eight cases (66.7%), the liver enzymes and bromosulphthalein clearance indicated a significant degree of liver dysfunction. In 1971, alteration of hepatic function was detected in nine cases (18%) evaluated via the bromosulphthalein test, and in three cases (6%) evaluated via elevated ALT, among 50 chronic marijuana users (two cigarettes of 0.5 g each/day), but it was later discovered that they were also alcohol consumers. Bromosulphthalein and ALT returned to normal values after a period of alcohol withdrawal, under continued use of marijuana.^21^ A study on users of various types of drugs (heroin, marijuana and hashish), taken parenterally or non-parenter-ally, revealed that 66% and 41% of the patients, respectively, showed high levels of AST. Liver biopsies revealed that 53% of the cases showed the classical aspects of acute viral hepatitis and 44% the characteristics of nonspecific reactive hepatitis, while there was no evidence of increased connective tissue.^22^

Our results indicated that the fraction of users of marijuana alone, out of the whole sample of drug users, was smaller than that of users of associations of marijuana with other drugs. The results also indicated that associations with crack or alcohol were initiated about 1 year after the use of marijuana alone, which is consistent with other observations^3,23^ that marijuana is usually the drug with which usage starts. The mean age of users of marijuana in association with crack was significantly lower than for consumers of marijuana in association with alcohol. In other Brazilian series, the prevalence of marijuana consumption among cocaine-addicted populations was 33%^24^ and 38.75%,^25^ but in ours the association with crack reached 67.5%. These observations should be taken into consideration by those postulating liberation of marijuana for recreational or medicinal use.

Absence of some clinical signs, especially jaundice, as demonstrated here, could be among the reasons for the neglect in evaluating hepatic damage among marijuana users, in studies of the effects of the drug on the organ system.^4^ The non-correlated liver enlargement and enzyme alterations, in the marijuana group, could suggest that some cases of liver enlargement were consequent not on parenchymal lesion but rather on cell hyper-trophy/hyperplasia, especially the Kupffer component. This hypothesis seems reinforced by the high prevalence of splenomegaly, again not correlated with alterations in liver enzymes or signs of portal hypertension. The correlation between liver palpation characteristics and histopathological alterations^26,27^ would suggest that, among marijuana users, the liver alterations were only of degenerative type in 40%, and inflammatory or degenerative/inflammatory type in 33.3% of the cases. The possibility of the occurrence of fibrosis, indicated by palpation, was indicated in little more than a quarter of the cases.

Our observations regarding spleen enlargement in the marijuana group, detectable only through percussion in Traube's space, are not consistent with the report on spleen shrinkage in rodents submitted to high doses of cannabinoids.^28^ The significance of cannabinoid receptors identified in macrophages at the marginal zone of the spleen^29^ and in blood lymphocytes^30^ remains obscure.

Alterations in AST, ALT and AP among users of marijuana alone were usually slight, in spite of being frequent, and were found respectively in 42.3% of cases, 47 – 114 U/ml; 34.6%, 38-124 U/ml; and 53.8%, 61 – 110 UI/l. The high prevalence of alterations in aminotransferases in the marijuana/alcohol group (AST, 78.6%; ALT, 71.4%) could be interpreted as synergic enhancement of the toxicity of both drugs.

The raising of AP, which was slight but consistent in all groups, could at least in part reflect defective bile excretion due to bile channel lesion. Divergent behavior of AP and GGT, the latter elevated only in the marijuana/alcohol group, could result from induction of GGT by alcohol^31^ or derive from the two enzymes being produced at different sites.^32^

Analyses in studies on the hepatotoxicity of marijuana or cannabinoids should take into consideration various factors, detailed below.

- The diversity of composition and potency of the preparations, according to the geographic origin of cultivars and the plant organs utilized. The marijuana utilized by our patients was in the form of dried macerated inflorescence, leaves, sprouts and stems, so that the delta-9-tetrahydrocannabinol concentration could vary according to the proportions of the parts. In samples from two different batches seized by the São Paulo State Police, the delta-9-tetrahydrocannabinol content measured by gas chromatography was 0.82 and 2.2%.^33^ The concentration of delta-9-tetrahydrocannabinol in marijuana has increased significantly over recent decades, from 1 - 5% up to 10 - 15%.^13,34^ Such increases in potency could amplify the risks of more serious liver affections, if marijuana were to display dose-related behavior similar to what is observed in relation to alcohol.
- The pattern of drug usage, especially with regard to the duration of the habit and daily amount consumed. The marijuana users in this study started the habit earlier than in another Brazilian study^1^ (13.6 rather than 15.2 years of age) and had used the drug for about five years. The frequency of use and daily dose were also higher than in another study evaluating the toxicity of the drug,^21^ which could explain some of the differences in results.
- Nutritional states or alterations that could favor liver damage become especially relevant in small patient series. Hypoalbuminemia is considered to be a useful indicator of nutritional deficit and this was present in 23, 19 and 36% of the cases in the respective groups of marijuana, marijuana/crack and marijuana/alcohol users. In the first group, there was no correlation between serum albumin levels and those of liver enzymes, especially those of ALT, thus suggesting that malnutrition should not have been of influence in determining susceptibility to the drug.
- In the general population, the use of illicit drugs through non-parenteral routes is associated with infection by viruses causing hepatopathy, especially the hepatitis B and C viruses. The interference of such infections was minimized by excluding from this study homosexuals, parenteral drug users, and patients that had piercings or reported having received acupuncture, blood derivatives or blood transfusion. The patients included in this study aspirated marijuana via cigarettes or crack via orally smoked pipes. In this way, the possibility of viral contamination would be restricted to the oral, sexual^35^ or skin decoration routes.^36^ The possibility of transmission of the hepatitis B virus through the saliva is still debatable, while transmission of the C virus has not been indicated.^37,38^ Association of the hepatitis C virus (HCV) with cocaine usage was related to inhalation from collective intra-nasal straws, suggesting that the contamination was really parenteral, through the bruised nasal mucous membrane.^36,39,40^

Usage of drugs as a risk factor for sexually transmitted diseases has been frequently taken for granted but should be better evaluated. Marijuana is known not to be an aphrodisiac,^41^ which could be related to testosterone reduction,^42^ and frequent periods of absence of sexual desire have been reported by chronic cocaine users.^43^ The sexual transmission of HCV is controversial, and has been considered inefficient in Unites States surveys.^36^ In Brazil, the evidence for this has been negative^44^ or small (8.8%).^45^ The rate of chronic disease after acute B-virus hepatitis was 1.7%^46^ and the overall frequency of HBsAg in nonparenteral drug users 1.6%,^41^ while the prevalence of anti-HCV, among such users, especially of crack, has ranged from 21% (in New York) to 2% (San Francisco and Miami).^47^

The prevalence of liver enzyme alterations in marijuana users was higher than would be expected under the unrealistic hypothesis that all patients were bearers of viral infection showing enzyme alteration. The mean levels of the AST:ALT ratio were high in all groups (marijuana, 1.47; marijuana/crack, 1.48; marijuana/alcohol, 1.34). Within the setting of viral hepatitis, the AST:ALT ratio is typically less than 1.0.^48^

Furthermore, tattooing was investigated, which is a trend among white and brownish male drug users and a form of virus transmission. Comparisons of the prevalence of liver alterations and the enzyme levels in tattooed and non-tattooed subgroups of the marijuana group revealed that the latter showed higher prevalence and levels of aminotransferases, especially ALT. This indicates that liver alterations observed were most probably due to marijuana itself, rather than a possible associated viral infection.

The results point towards the occurrence of possible marijuana hepatotoxicity. This should be taken into consideration along with other organ affections resulting from chronic marijuana usage, in discussions on its legalization and therapeutic uses.
